# Thoracic Aortic Aneurysms

**Published:** 1908-03-28

**Authors:** Dyce Duckworth

**Affiliations:** Consulting Physician to St. Bartholomew's Hospital; Senior Physician to the Seamen's Hospital, Greenwich.


### Hospital Clinics.

### THORACIC AORTIC ANEURYSMS.

By Sir DYCE DUCKWORTH, M.D., LL.D., Consulting Physician to St. Bartholomew's Hospital; Senior Physician to the Seamen's Hospital, Greenwich.

A Clinical Lecture delivered at the London School of Clinical Medicine.

The subject of my lecture to-day is a very large one to discuss in one sitting. I can, therefore, only take up some parts of it, and speak of them mainly from a clinical standpoint. I assume that you are sufficiently acquainted with the intimate pathology of aneurysms, and have already discovered for yourselves that they occur almost exclusively in the male sex, and that their ætiology is practically dependent upon a degenerative condition of the coats of the arterial tunics. The main causes of this degeneration are limited to three or four agencies. First, I will mention a tendency or textural proclivity to premature decay in the whole arterial system. This is probably inherited as a constitutional delicacy. Next we find as special determining causes, the influence of strain, long-continued; the mischief wrought by over-indulgence in alcoholic beverages; and the vicious effects of constitutional syphilis which are well recognised as among the most potent destroyers of arteries. All, or several, of these causes are found to be conjoined in the large majority of cases of aneurysm. To these we might add as an occasional cause the influence of lead-impregnation, which falls heavily on the arterial tunics throughout the body. You may note that the occurrence of heart disease of any kind, whether in the common forms of endocarditis, valvular changes, or of pericarditis, is not among the causes of aneurysm as generally met with. Further, the occurrence of even large aortic aneurysms has no special effect on the condition of the myocardium. You might expect this, but you will not find changes in the direction of hypertrophy or dilatation of the cardiac walls, unless, indeed, there be combined with the aneurysm some valvular lesion, more especially in the aortic segments. This is a fact not generally recognised. The heart may be displaced by a large aneurysm, and so give the impression of enlargement.

In this clinic we are apt to meet with numerous examples of the disease as occurring in seamen. Yet the occurrence is certainly more common in soldiers. In both cases we find the element of strain, and in both, alas! we too often find the other malign elements of alcoholic excess and syphilis. The greater frequency in soldiers is explained by the influence of their dress and the compression by equipments while making prolonged exertion; the sailor being loosely draped and working under easier conditions. The more open-air life of the sailor, and his temporary enforced abstention from alcohol, probably count for something as against the barrack and urban life of the soldier.

As I have said, aneurysms are met with chiefly in males. When they occur in women we commonly find that they have been exposed to strain, indulgence in alcohol, and almost always the victims of syphilis. Our museum here has a collection of morbid specimens illustrating this subject in a manner hardly to be equalled elsewhere. I think that aneurysms are perhaps less frequently met with now than they were formerly. Improvements in dress, in morals, and a better treatment of syphilis appear to be telling favourably in this respect. We may declare that, given no special textural proclivity to arterial decay, aneurysms ought to be rarely met with if the ordinary causes of it were removed. They are comparatively uncommon in the upper ranks of society.

I note next that there is what may be termed an aneurysmal age, the disease commonly arising at the end of the third or early in the fourth decade, thirty-nine being about an average age for its occurrence. We meet with it, however, in later periods. The aneurysmal age is also the age for the worst manifestations of late syphilis in cases inadequately treated at the outset.

Clinically, we have to recognise two main varieties of aortic aneurysm: those with symptoms and physical signs, and those with symptoms and few or no physical signs. The last are apt to be undetected during life; at least they were more difficult of detection until we were able to secure indications of their presence by skiagraphy, which has proved of great service in diagnosis of obscure cases. Thus it is that the diagnosis in a given case may either be very easy or very difficult. They either discover themselves or may remain obscure till a sudden fatal issue with profuse internal or external hæmorrhage occurs. Take the ordinary symptoms in the case of the different parts of the thoracic aorta. The intima and middle coats of the vessel yield in time to the pressure of the blood column, pumped onwards, say 100,000 times each day, and form a tumour which expands with each pulsation, tending gradually to increase centrifugally. Here there is a definite intruding source of pressure upon all adjacent parts. Your anatomical knowledge tells you what these are. This pressure is resented, and gives tokens of its presence by pain, increased pulsation, delay in circulation, venous plethora, dyspnœa, tracheal irritation, a characteristic clanging, noisy cough, and by the occurrence of a murmur of a blowing or rasping quality, systolic in time, heard over the newly formed tumour. A significant physical sign of aortic aneurysm is the occurrence of a loud second sound in the aortic area. This is of particular value in obscure cases where there is no systolic bruit audible, owing to density and large amount of laminated clot in the tumour. I cannot now refer to the several varieties of aneurysm which are met with. It must suffice to mention the true and the false, the former commonly described as fusiform, where the dilatation involves the vessel uniformly, or may form a sac or pouch; the latter in which there is rupture of the vessel and the aneurysm is formed among the adjacent tissues. The dissecting variety is rather of the nature of an accident, due to rupture of the intima and a separation of the other tunics. The differential diagnosis of these varieties is not commonly made during life. The varieties known as embolic and infective must not be considered to-day.

The most frequent position is found to involve the ascending and transverse portions of the aortic arch. If the tumour is small and at the beginning of the ascending portion, there may be difficulty in deciding whether there be an aneurysm or only a degeneration of the aorta. Death may be sudden if such a tumour bursts, as it commonly does, into the pericardial sac. In the case of the ascending and transverse portions, we find a progressing swelling, exercising pressure on all adjacent structures, especially the vena cava, and pushing its way forwards or backwards, compressing the right or left recurrent laryngeal nerves, the sympathetic nerve, and sometimes the thoracic duct. We judge of its progress by the several structures it successively involves. The size of these tumours is sometimes enormous, and the heart may be much displaced by them. As they push forwards we find a gradual prominence over the thorax with visible and palpable pulsation in it, commonly above the third rib and to the right of the sternum. The sign of tracheal tugging may be present. The pulses may be unequal at the wrist, and a pulsation is often felt at the sternal notch. When the descending portion of the arch is involved the sac presses backward and to the left side, causing erosion of the vertebræ from the third to the sixth, and inducing intense pain, dysphagia, and pressure on the left bronchus. In the descending thoracic aorta the sac is generally found low down, eroding the vertebræ and forming a tumour inside the left scapula. When near the surface, or prominent, we find dullness to percussion. If the aneurysm is small and seated deeply, there is no alteration of percussion note. A prominent pulsating tumour with a heaving and expansile impulse can be nothing else than an aneurysm, and if we find a palpable diastolic shock following the impulse we may be sure of the nature of the case.

The pain is sometimes severe, especially if vertebral erosion is in progress. There may be anginal attacks in the case of aneurysm of the first part of the ascending aorta, radiating towards the left shoulder and down the arm. The "brassy" cough indicates pressure on the recurrent laryngeal nerve, and with this there may be compression of the trachea or the left bronchus and rentention of secretion, with fever. Small hæmoptyses are significant in association with these symptoms, preceding a larger and fatal one from rupture of the tumour, more often in the case of aneurysms of the third part of the arch and its descending portion.

Dysphagia may supervene, and we are careful to abstain from passing a bougie in such cases. Clubbing of the fingers of one hand may occur. This is always a sign of some obstruction in the thorax, and has been attributed by Dr. Payne to blocking of lymphatics. Inequality of the pupils indicates pressure on the sympathetic nerve. Compression of the thoracic duct entails a general failure in nutrition which is sometimes to be noted.

The prognosis is always grave. We speak with bated breath of *cure* of thoracic and abdominal aortic aneurysms; yet cures are not unknown. Clotting and consolidation of the fibrinous contents may occur and seal up the tumour. This is most likely to happen in sacculated aneurysms. There may be long progress in some cases, but that progress is too commonly in an untoward direction. We can promote delay, and secure great alleviation by treatment. The course of the disease may be protracted for years, to ten years or more even, in the cases of large tumours. Much depends on the amenability of the patient, and his submission to persistent and appropriate treatment. Some of the cures have only been discovered at autopsies where the disease has been unrecognised during life.

In treatment rest is the essential element, with freedom from all excitement of the circulation, so that the numbers of the cardiac contractions are materially reduced. The main endeavour is to promote clotting and obliteration of the sac. A reduced diet and restriction of liquids are essential. This condition is sometimes intolerable after a few weeks, and we have always to treat patients and not diseases. The plan of treatment recommended by Mr. Tufnell, a Dublin surgeon, some forty years ago, has been more or less adopted during that period. Rest and restricted allowance of food and drink are the main features of it. He prescribed about 8 oz. of solid and 8 oz. of liquid in the day. This is a starvation dietary, and cannot long be maintained. It must never be carried out if there is any aortic incompetence. The quantities just mentioned may be safely increased in most cases. The volume of blood is diminished, and the arterial tension within the sac is lessened. Coagulation is thereby promoted. It is only applicable for a few weeks, and is specially indicated for sacculated tumours. Milk and Bordeaux red wine are the best liquids to employ. Thirst and constipation are two of the outward effects of this plan, especially in warm weather. The benefits are certainly appreciable, but cannot be regarded as more than palliative in most cases, and we generally have to suspend this extreme method, and permit a moderate and mixed diet, with gentle exercise as far as it can be borne.

In regard to medicines, the use of iodide of potassium in full doses, as recommended by George Balfour, of Edinburgh, is most deservedly in favour: 15 to 30 grains thrice a day may be given in decoctions of sarsaparilla. It probably lowers the blood pressure and aids in limiting and consolidating the coats of the tumour. I can say nothing in favour of any surgical interference with the aneurysm itself. Many and varied methods have been carried out in the direction, including electrolysis, packing with wire, horse hair, or silk thread. Suffice it to say, that I have never felt justified in urging any one of these. My colleague, Dr. Guthrie Rankin, has employed the method of introducing gelatine subcutaneously, as suggested by Lancereaux, of Paris, ten years ago, with some measure of success in reducing the size and activity of aneurysms. Ordinary treatment was carried on simultaneously. Fifteen to twenty grains of pure gelatine were injected in fifty or one hundred cubic centimetres of sterilised saline solution into the thigh or pectoral region.* Twenty of such injections at intervals may be required. Venesection may be called for to relieve venous engorgement and cyanosis with dyspnœa, and is useful.

When the sac pushes externally, as it is apt to do in the front of the chest, it becomes necessary to fortify the integuments over the tumour. I have used styptic colloid with advantage in such cases, also solution of guttapercha in benzole. The skin breaks at last, and oozing of blood occurs. Solution of perchloride of iron (strong) on lint is then an appropriate application, and a moulded shield of guttapercha may be adapted to secure support, or an elastic bag, as recommended by Dr. Osler, may be bound over the tumour. All these measures are but of temporary use. The tumour bursts at last, and the hæmorrhage is then copious and fatal. We may resort to morphine hypodermically to allay the misery of the final stages of aneurysm and so promote euthanasia.

There are no other drugs which prove of much service in delaying the progress or promoting an arrest of the disease. Aconite may be mentioned, and a trial of chloride of calcium is indicated as tending to promote coagulation in the sac. A quiet, easy life, restricted solid and liquid food, potassium iodide, with attention to the bowels, constitute the main features of the service we can render to these unfortunate sufferers.

* *Med. Chirurg. Trans.* vol. 86, 1903, p. 376.

